# Hypoxemia after thoracic aortic aneurysm repair

**DOI:** 10.1016/j.xjon.2026.101808

**Published:** 2026-04-10

**Authors:** Gift Owolabi, Yuichiro Kitada, Jack Nickles, Yanling Zhao, Paul Kurlansky, Lauren Sutherland, Jonathan Hastie, Thomas O'Donnell, Virendra Patel, Adham Elmously, Hiroo Takayama

**Affiliations:** a Division of Cardiac, Thoracic, and Vascular Surgery, NewYork-Presbyterian Hospital/Columbia University Irving Medical Center, New York, NY; b Center for Innovation and Outcomes Research, NewYork-Presbyterian Hospital/Columbia University Irving Medical Center, New York, NY; c Department of Anesthesiology, NewYork-Presbyterian Hospital/Columbia University Irving Medical Center, New York, NY

**Keywords:** aneurysm repair, hypoxemia, circulatory arrest, lower body ischemia

## Abstract

**Objective:**

To characterize early postoperative hypoxemia (POH) after proximal aortic aneurysm repair, evaluate its association with outcomes, and determine whether circulatory arrest (CA) contributes to the development of POH.

**Methods:**

We performed a single-center retrospective study of 790 adults undergoing proximal aortic aneurysm repair via sternotomy (2012-2024). POH was defined as an arterial oxygen tension/inspired oxygen fraction ratio <300 mm Hg at 6 hours and categorized as no hypoxemia (>300), mild (200-300), or moderate/severe (<200). Baseline and operative characteristics were balanced using 3-way inverse probability of treatment weighting. The primary end point was composite respiratory morbidity (including prolonged ventilation and supplemental oxygen use, reintubation, and postoperative pneumonia); secondary end points included 7-year mortality. Weighted regression evaluated factors associated with POH, including CA exposure.

**Results:**

POH occurred in 433 patients (54.8%): 266 (33.7%) mild and 167 (21.1%) moderate/severe; 357 (45.2%) had no hypoxemia. After inverse probability of treatment weighting was performed, the severity of hypoxemia was associated with composite respiratory morbidity (43.8% none vs 50.6% mild vs 57.0% moderate/severe; *P* = .030). However, hypoxemia was not associated with 7-year mortality (hazard ratio, 0.77; 95% CI, 0.38-1.37; *P* = .321). Use of CA was not associated with moderate/severe POH (odds ratio, 0.96; CI, 0.60-1.53; *P* = .864).

**Conclusions:**

Early POH is common after proximal aneurysm repair and is associated with increased respiratory morbidity but not mortality. CA does not appear to contribute to moderate/severe POH.

## Graphical Abstract

Early POH (PF ratio at 6 hours) is common after open thoracic aortic aneurysm repair. Increasing hypoxemia severity is associated with greater morbidity (ventilation duration, supplemental oxygen requirements, ICU length of stay, ICU readmission, and pneumonia) but not early mortality or long-term survival. After IPTW, circulatory arrest was not associated with moderate/severe hypoxemia (PF < 200). Overall, circulatory arrest and clinically typical arrest durations do not appear to meaningfully contribute to moderate/severe early POH. *POH*, Postoperative hypoxemia; *PF*, arterial oxygen tension/inspired oxygen fraction ratio; *ICU*, intensive care unit; *IPTW*, inverse probability of treatment weighting; *TAA*, thoracic aortic aneurysm; *ARDS*, acute respiratory distress syndrome.

**Figure:**
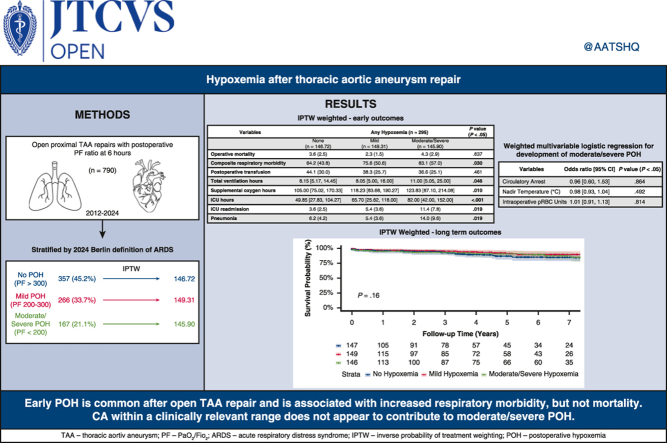

**Figure:**
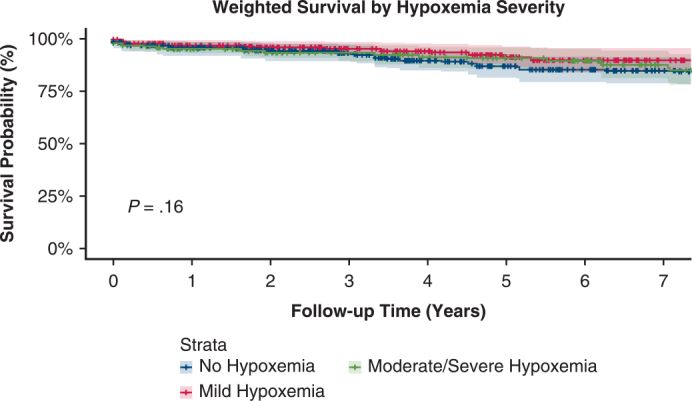
Early postoperative hypoxemia is not associated with 7-year mortality.

**Central Message**

Severity of postoperative hypoxemia was associated with respiratory morbidity, whereas circulatory arrest use and duration were not linked to moderate-to-severe hypoxemia after thoracic aortic repair.

**Perspective**

Previous studies evaluating the relationship between circulatory arrest and postoperative hypoxemia focus largely on ATAAD and report conflicting findings. This study examines a contemporary cohort with thoracic aortic aneurysm and provides a granular, risk-adjusted analysis of arrest use and duration, helping clarify their limited contribution to clinically significant postoperative hypoxemia.

Postoperative hypoxemia (POH) is a common complication after cardiopulmonary bypass (CPB), with reported incidences ranging from 25 to 50%^1^, ^2^, ^3^ across cardiac surgical populations. Although POH is often managed with supplemental oxygen, it has been independently associated with increased short-term mortality, prolonged stays in the intensive care unit (ICU) and hospital, and greater health care costs.^2^^,^^4^^,^^5^ Beyond its acute manifestations, CPB-associated POH may lead to persistent structural and functional pulmonary changes—including reductions in vital capacity and residual volume—suggesting potential long-term consequences that remain incompletely characterized.^6^

Established risk factors for POH include obesity, advanced age, preoperative chronic obstructive pulmonary disease, impaired left ventricular function, and prolonged CPB duration.^2^^,^^5^^,^^7^ The contribution of CPB itself is well described: longer bypass times amplify the systemic inflammatory response and exacerbate ischemia–reperfusion injury after periods of reduced or absent pulmonary blood flow.^8^^,^^9^

Thoracic aortic operations represent a particularly high-risk surgical context, with reported POH incidences approaching 68%.^10^ Consequently, predictors of POH have been extensively studied in acute type A aortic dissection (ATAAD), whereas data in patients undergoing surgery for chronic aortic disease remain comparatively sparse.^1^^,^^3^^,^^10^, ^11^, ^12^, ^13^ A distinguishing feature of aortic surgery is the frequent use of circulatory arrest (CA), which may impose pulmonary injury beyond that attributable to standard CPB alone.^14^ However, the independent and quantitative effects of CA, particularly lower-body ischemia (LBI) duration, on postoperative pulmonary function in chronic aortic disease are not well defined.

Conventional reasoning suggests that longer ischemic intervals during CA would result in greater lung injury, yet existing studies offer conflicting findings, with some suggesting minimal impact of CA on the need for postoperative respiratory support.^15^ In addition, the depth of and duration of hypothermia during CA have been proposed as potential modifiers of pulmonary risk, with emerging evidence that short and mild hypothermia may be less injurious than prolonged moderate hypothermia.^15^^,^^16^ Collectively, these inconsistencies highlight a critical knowledge gap in understanding how CA characteristics influence POH after thoracic aortic surgery.

In this study, we sought to evaluate the association between CA-related variables and POH and to examine the downstream clinical implications of POH. We hypothesized that the use of CA and longer LBI durations would be associated with an increased risk of POH at 6 hours postoperatively and that POH would, in turn, be associated with worse long-term mortality.

## Methods

### Study Design and Patient Selection

The institutional review board of Columbia University Medical Center approved the study protocol and publication of data (no. AAAR2949; most recent approval date: April 3, 2025). Patient written consent for the publication of the study data was waived by the institutional review board, given that all data were retrospectively collected. We conducted a single-center study of adult patients who underwent open proximal thoracic aortic aneurysm repair at our institution between 2012 and 2024.

**Figure E1:**
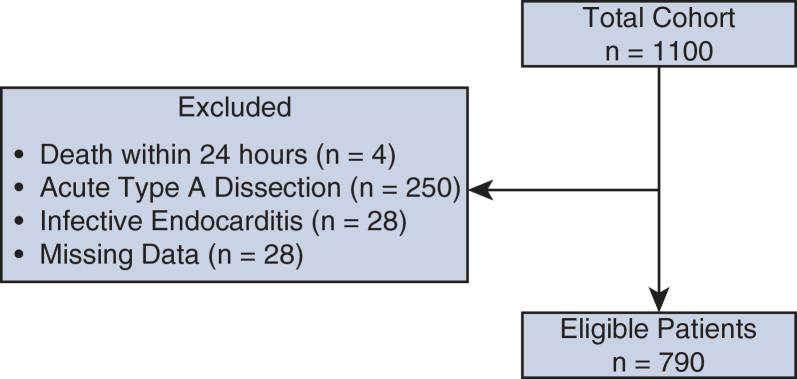
Consolidated Standards of Reporting Trials diagram. A total of 310 patients were excluded according to inclusion and exclusion criteria (n = 282) and missing data (n = 28). Some patients had overlapping exclusion criteria. The final cohort size was 790 patients.

Eligible patients were aged 18 years or older and underwent surgical aneurysm repair involving the proximal aorta, defined as the aortic segment from aortic root to arch (zone 3), via median sternotomy. Exclusion criteria (Figure E1) included preoperative oxygen dependence, surgical indication of ATAAD or infective endocarditis, and missing outcome data. Four patients who died within 24 hours of their operation were also excluded to avoid confounding of postoperative respiratory status.

POH was defined as an arterial oxygen tension/inspired oxygen fraction (Fio_2_) (PF) ratio <300 mm Hg measured closest to 6 hours postoperatively and classified as mild (200-300), moderate (100-200), or severe (<100), consistent with the PF ratio thresholds of the 2024 Berlin definition of acute respiratory distress syndrome.^17^ A 6-hour time point was selected on the basis of previous literature, identifying this as a consistently clinically meaningful early postoperative period when gas exchange abnormalities are most pronounced^1^^,^^5^^,^^10^ and because it aligns with typical early extubation benchmarks in cardiac surgery.^18^ We selected a PF ratio threshold of <200 (moderate acute respiratory distress syndrome) for focused analyses on the basis of previous cardiac surgery literature indicating that this cutoff more reliably reflects clinically significant hypoxemia rather than transient, low-grade postoperative changes.^1^^,^^2^^,^^11^^,^^19^^,^^20^ The primary end point was composite respiratory morbidity, defined as prolonged invasive ventilation (Society of Thoracic Surgeons definition ≥24 hours), supplemental oxygen use ≥120 hours, reintubation, or postoperative pneumonia. In the absence of a consensus definition for prolonged supplemental oxygen use, a previous study has shown ≥120 hours to be associated with increased mortality in patients who undergo cardiac surgery.^21^ Secondary end points included 7-year all-cause mortality and in-hospital outcomes (including the individual components of the primary end point): operative mortality, postoperative transfusion, total ventilation hours, reintubation, supplemental oxygen duration, return to operating room for rebleeding or another reason, ICU hours, ICU readmission, sternal wound infection, postoperative pneumonia, effusion, stroke, and length of stay. Additional analyses were conducted to understand the factors, particularly CA, that are associated with POH. PF ratios were calculated by matching arterial oxygen tension values from arterial blood gas measurements with concurrent Fio_2_ recordings. Fio_2_ values were obtained directly from the electronic medical record as documented in the respiratory flowsheet at the time of arterial blood gas sampling. These values reflect device-recorded or clinically charted Fio_2_ levels corresponding to the oxygen delivery modality in use (eg, ventilator, high-flow nasal cannula, Venturi mask, or low-flow nasal cannula). No additional Fio_2_ adjustments or estimations were applied by the study team. Although a 6-hour postoperative time point was targeted, a ±3-hour window was permitted to account for intermittent sampling, with the value closest to 6 hours preferentially selected.

Preoperative demographics, procedural characteristics, and postoperative complications were collected by reviewing the electronic medical record. Whenever able, we defined complications based on the Society of Thoracic Surgeon Adult Cardiac Database Version 4.20.2. Long-term survival status was collected through clinical encounters as well as phone calls to patients and referring physicians. Death information through December 31, 2022, was supplemented by querying the National Death Index,^22^ a centralized database of death records compiled from state vital statistics offices established by the Centers for Disease Control and Prevention's National Center for Health Statistics.

LBI was defined as the period of CA in which blood flow to end organs, excluding the brain, was interrupted. Patients who did not undergo CA were assigned a duration of 0 minutes and were included in all analyses except for analysis of the only CA subgroup.

### Intraoperative Management

Our intraoperative procedural and CPB management has been previously reported.^23^ The site of arterial cannulation was at the discretion of the attending surgeon on the basis of preference, patient anatomy, and pathology, and incidence of site varied as such. Surgical indication was determined by the attending surgeon, on the basis of most recent American Heart Association, American College of Cardiology, and European Society of Cardiology Guidelines.^24^ For aortic root replacement, the aortic valve was spared with reimplantation technique whenever appropriate, as previously described.^25^, ^26^, ^27^, ^28^ When replacement was necessary, the prosthetic valve was chosen on the basis of the American Heart Association/American College of Cardiology and European Society of Cardiology Guidelines as well as patient preference. Management of CPB was standard for our study period. Our institution's standard surgical management has been described by Yamabe and colleagues.^25^ After cannulation, crystalloid in the bypass circuit is replaced with patient's blood with retrograde autologous priming as tolerated. Packed red blood cell (pRBC) transfusion is discussed with hematocrit level of below 21. Standard bypass parameters were mild hypothermia (32 °C) with a pump flow rate of 2.5 mL/cm^2^ and goal mean arterial pressure of 60 to 80 mm Hg. DelNido cardioplegia is used.^29^ Distal aortic anastomosis for arch replacement was performed under hypothermic CA. Most hemiarch replacements were conducted using retrograde cerebral perfusion alone at 20 °C without antegrade cerebral perfusion (ACP). In contrast, total arch replacements were performed with bilateral ACP at 24 °C. During the distal anastomosis, distal systemic perfusion (including renal perfusion) was temporarily halted and then reinitiated via the side arm of the aortic graft upon completion of the anastomosis. For patients requiring ACP, perfusion was delivered at 8 to 12 mL/kg/min either through an axillary cannula with clamping at the base of the innominate artery, or by direct cannulation of the innominate and/or left common carotid artery using balloon-tip catheters. When central aortic cannulation was used, a brief period (1-2 minutes) of retrograde cerebral perfusion via the superior vena cava was instituted at the onset of CA to prevent air embolism while the distal arch and branch ostia were exposed for cannulation. Bilateral ACP was routinely used for partial or total arch replacement. The supra-aortic vessels were then individually reconstructed using a multi-branch graft.

### Statistical Analysis

Normality of continuous data were assessed using the Shapiro-Wilk test. Continuous variables were additionally evaluated for distributional characteristics and potential outliers using graphical inspection and interquartile range methods. Values identified as statistical outliers were reviewed for biological plausibility; no implausible values were identified after data verification, and clinically plausible extreme values were retained in analyses. Categorical variables were reported as counts and percentages; continuous variables were reported as mean ± standard deviation when normally distributed and as medians with interquartile ranges otherwise. For 2-group comparisons, we used the Pearson χ^2^ or Fisher exact test for categorical variables and the Student *t* test or Mann-Whitney *U* test for continuous variables. When POH was analyzed across 3 severity groups (none, mild, moderate/severe), omnibus differences were first tested with the one-way analysis of variance or Kruskal-Wallis test (continuous) or the Pearson χ^2^ or Fisher exact test (categorical); if the omnibus test was significant (*P* < .05), pairwise Student *t* test or Mann-Whitney *U* (continuous) or pairwise χ^2^/Fisher exact tests (categorical) were performed with Bonferroni correction for multiple comparisons.

**Figure E2:**
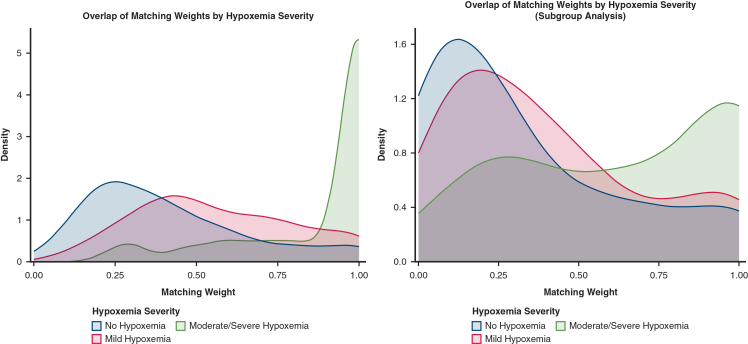
Overlap of matching weights by hypoxemia severity. Weights were normalized to the 0 to 1 range to improve overlap among the 3 hypoxemia groups.

To account for baseline differences among POH severity groups, the inverse probability of treatment weighting (IPTW) method was performed to estimate propensity scores using a multinominal logistic regression model including baseline demographic and intraoperative variables, such as age; body mass index; ethnicity; smoking status; hypertension; chronic obstructive pulmonary disease; estimated glomerular filtration rate; preoperative myocardial infarction; reintervention (previous percutaneous coronary intervention or operation); preoperative hemoglobin, albumin, and platelet counts; surgical indication; operation; arterial cannulation site; CPB time; and intraoperative pRBC transfusion. Intraoperative variables were included to balance operative complexity and physiologic burden across POH severity groups, because these factors precede POH ascertainment and may influence both POH severity and postoperative outcomes. CA-related variables (including CA use and duration) were intentionally not included in the propensity model and were subsequently evaluated as predictors of POH severity in weighted regression analyses. Weights were constructed on the basis of propensity scores^30^ and were applied to generate a weighted pseudo-population with balanced covariates across POH severity groups. Weights were normalized to the 0 to 1 range to improve overlap among the 3 treatment groups (Figure E2). Covariate balance was assessed using standardized mean differences, with values <.10 indicating adequate balance. Given limited power in subgroup analyses, residual imbalance (standardized mean difference, 0.10-0.20) was tolerated for covariates judged unlikely to materially influence outcomes.

Multivariable regression analyses were performed to assess associations between covariates and outcomes using IPTW-weighted samples. Models were fit using the survey package in R to obtain robust (sandwich) variance estimates. Specifically, tables were used to compare in-hospital outcomes; Cox regression models were used to assess 7-year all-cause mortality; logistic regression models were used to assess moderate/severe POH; and linear regression modeling was used to assess continuous PF ratio. A primary Cox model was used to estimate the association between POH and mortality in the weighted cohort without adjustment for postoperative complications (total association). A secondary model additionally adjusted for postoperative complications to explore whether associations persisted independent of downstream events. The proportional hazards assumption was assessed using Schoenfeld residuals in weighted Cox models. Covariates for all multivariable models were selected on the basis of clinical grounds and from univariate screens (*P* < .10). Multicollinearity was assessed with variance inflation factors, ensuring that a cutoff value of 5 was met. Weighted Kaplan–Meier survival curves were plotted to illustrate survival. To explore potential nonlinearity between CA duration and POH, we modeled the adjusted odds of any POH using restricted cubic splines with 4 knots. All statistical analyses were performed using R statistical software, version 4.5.0.

## Results

### Baseline Characteristics and Operative Details

From an original cohort of 1100 patients, there were 790 (71.8%) patients included in the study, after the exclusion of 310 (28.2%) patients, including 4 patients who died within 24 hours, 250 and 28 patients with surgical indication for acute type A dissection and infective endocarditis, respectively, and 28 patients excluded for missing outcome data (Figure E1).

Baseline characteristics are described in Table 1. The median age of patients with and without POH was both 63.0 years (*P* = .328), and both groups mainly were composed of male patients (*P* = .397). Patients with POH had a greater median body mass index (29.00 vs 26.85 kg/m^2^, *P* < .001). Patients with POH had a greater incidence of hypertension (77.1% vs 70.0%, *P* = .029) but comparable rates of lifetime smoking (43.2% vs 37.0%, *P* = .089), chronic obstructive pulmonary disease (12.0% vs 10.6%, *P* = .625), heart failure (43% vs 44%, *P* = .727), and reintervention (30.9% vs 27.5%, *P* = .320) with patients without POH.

**Table 1:** Baseline characteristics and risk factors

Variables	Overall, n = 790	No hypoxemia, n = 357	Hypoxemia, n = 433	*P* value
Demographics				
Mean age, y, median [IQR]	63.00 [53.00, 71.00]	63.00 [53.00, 70.00]	63.00 [54.00, 71.00]	.328
Female, n (%)	148 (18.7)	72 (20.2)	76 (17.6)	.397
BMI, kg/m^2^, median [IQR]	27.92 [25.04, 31.70]	26.85 [23.77, 30.12]	29.00 [25.89, 32.79]	**<.001**
Race				**.045**
White, n (%)	637 (80.6)	285 (79.8)	352 (81.3)	
Black/African American, n (%)	81 (10.3)	40 (11.2)	41 (9.5)	
Asian, n (%)	30 (3.8)	19 (5.3)	11 (2.5)	
Other, n (%)	42 (5.3)	13 (3.6)	29 (6.7)	
Ethnicity, n (%)	120 (15.2)	40 (11.2)	80 (18.5)	**.006**
Ever smoker, n (%)	319 (40.4)	132 (37.0)	187 (43.2)	.089
Emergent status, n (%)	6 (0.8)	4 (1.1)	2 (0.5)	.198
Risk factors				
Hypertension, n (%)	584 (73.9)	250 (70.0)	334 (77.1)	**.029**
Diabetes, n (%)	111 (14.1)	48 (13.4)	63 (14.5)	.733
Atrial fibrillation, n (%)	149 (18.9)	58 (16.2)	91 (21.0)	.106
Connective tissue disease, n (%)	7 (0.9)	5 (1.4)	2 (0.5)	.308
Coronary artery disease, n (%)	666 (84.3)	297 (83.2)	369 (85.2)	.496
Peripheral artery disease, n (%)	136 (17.2)	58 (16.2)	78 (18.0)	.575
CKD, n (%)	138 (17.5)	64 (17.9)	74 (17.1)	.830
eGFR, mL/min/1.73 m^2^, median [IQR]	81.91 [67.20, 93.26]	82.76 [68.41, 93.82]	81.41 [66.46, 92.51]	.286
COPD, n (%)	90 (11.4)	38 (10.6)	52 (12.0)	.625
CVD, n (%)	83 (10.5)	30 (8.4)	53 (12.2)	.102
MI, n (%)	43 (5.4)	15 (4.2)	28 (6.5)	.215
CVA, n (%)	45 (5.7)	17 (4.8)	28 (6.5)	.382
NHYA classification				.727
No heart failure, n (%)	447 (56.6)	200 (56.0)	247 (57.0)	
NYHA I, n (%)	122 (15.4)	50 (14.0)	72 (16.6)	
NYHA II, n (%)	96 (12.2)	48 (13.4)	48 (11.1)	
NYHA III, n (%)	113 (14.3)	53 (14.8)	60 (13.9)	
NYHA IV, n (%)	12 (1.5)	6 (1.7)	6 (1.4)	
Ejection fraction, median [IQR]	55.00 [53.00, 60.00]	57.00 [55.00, 60.00]	55.00 [52.00, 60.00]	**.050**
Reintervention, n (%)	232 (29.4)	98 (27.5)	134 (30.9)	.320
Surgical indication				.848
Aortic aneurysm, n (%)	768 (97.2)	348 (97.5)	420 (97.0)	
Chronic dissection, n (%)	22 (2.8)	9 (2.5)	13 (3.0)	

The overall cohort is presented and then divided into no hypoxemia versus hypoxemia (PF < 300) groups. Values in bold indicate significant *P* values. *IQR*, Interquartile range; *BMI*, body mass index; *CKD*, chronic kidney disease; *eGFR*, estimated glomerular filtration rate; *COPD*, chronic obstructive pulmonary disease; *CVD*, cardiovascular disease; *MI*, myocardial infarction; *CVA*, cerebrovascular accident; *NYHA*, New York Heart Association; *PF*, arterial oxygen tension/inspired oxygen fraction ratio.

Laboratory test results and procedural characteristics are described in Table 2. Patients with POH had a similar baseline hemoglobin level as patients without POH (14.0 vs 13.8 g/dL, *P* = .281). Otherwise, patients with POH had a lower incidence of concomitant aortic valve replacement (12.7% vs 18.2%, *P* = .022) compared with patients without but a similar incidence of isolated ascending aorta replacement (10.6% vs 11.8%, *P* = .694); aortic root replacement/repair (54.5% vs 55.2%, *P* = .537); aortic arch replacement (35.8% vs 35.3%, *P* = .319); and overall concomitant procedures (19.6% vs 16.2%, *P* = .256).

**Table 2:** Laboratory test results and procedural characteristics

Variable	Overall, n = 790	No hypoxemia, n = 357	Hypoxemia, n = 433	*P* value
Laboratory test results				
Hemoglobin, median [IQR]	13.90 [12.83, 14.90]	13.80 [12.90, 14.80]	14.00 [12.80, 14.90]	.281
WBC count, median [IQR]	6.82 [5.59, 8.16]	6.74 [5.35, 8.07]	6.87 [5.71, 8.19]	.156
Platelet count, median [IQR]	208 [174, 252]	213 [179, 251]	203 [171, 253]	.164
Albumin, median [IQR]	4.50 [4.20, 4.70]	4.50 [4.10, 4.70]	4.50 [4.20, 4.70]	.743
Hematocrit, median [IQR]	41.60 [38.70, 44.10]	40.80 [38.70, 43.80]	41.90 [38.60, 44.40]	.118
Creatinine, median [IQR]	1.00 [0.87, 1.16]	0.99 [0.86, 1.16]	1.00 [0.87, 1.16]	.351
Procedures				
Isolated ascending aorta replacement, n (%)	88 (11.1)	42 (11.8)	46 (10.6)	.694
Concomitant AVR/r w/o ARR/r				**.022**
Mechanical valve, n (%)	6 (0.8)	6 (1.7)	0 (0.0)	
Bioprosthetic valve, n (%)	107 (13.5)	56 (15.7)	51 (11.8)	
Repair, n (%)	7 (0.9)	3 (0.8)	4 (0.9)	
Aortic root replacement/repair				.537
VSRR, n (%)	126 (15.9)	53 (14.8)	73 (16.9)	
Mechanical Bentall, n (%)	26 (3.3)	15 (4.2)	11 (2.5)	
Bioprosthetic Bentall, n (%)	280 (35.4)	128 (35.9)	152 (35.1)	
Homograft, n (%)	1 (0.1)	1 (0.3)	0 (0.0)	
Aortic arch replacement				.319
Hemiarch, n (%)	165 (20.9)	81 (22.7)	84 (19.4)	
Partial arch, n (%)	67 (8.5)	28 (7.8)	39 (9.0)	
Total arch, n (%)	49 (6.2)	17 (4.8)	32 (7.4)	
Concomitant procedures, n (%)	143 (18.1)	58 (16.2)	85 (19.6)	.256
Concomitant CABG				.438
1-2 vessels, n (%)	73 (9.2)	29 (8.1)	44 (10.2)	
3+ vessels, n (%)	24 (3.0)	9 (2.5)	15 (3.5)	
Concomitant MVR/r				.227
Repair, n (%)	32 (4.1)	10 (2.8)	22 (5.1)	
Replacement, n (%)	15 (1.9)	8 (2.2)	7 (1.6)	
Concomitant TVR/r, n (%)	14 (1.8)	11 (3.1)	3 (0.7)	**.024**

The overall cohort is presented and then divided into no hypoxemia vs hypoxemia (PF <300) groups. Values in bold indicate significant *P* values. *IQR*, Interquartile range; *WBC*, white blood cell; *AVR/r*, aortic valve replacement/repair; *ARR/r*, aortic root replacement/repair; *VSRR*, valve-sparing root replacement; *CABG*, coronary artery bypass graft; *MVR/r*, mitral valve replacement/repair; *TVR/r*, tricuspid valve replacement/repair; *PF*, arterial oxygen tension/inspired oxygen fraction ratio.

**Figure E3:**
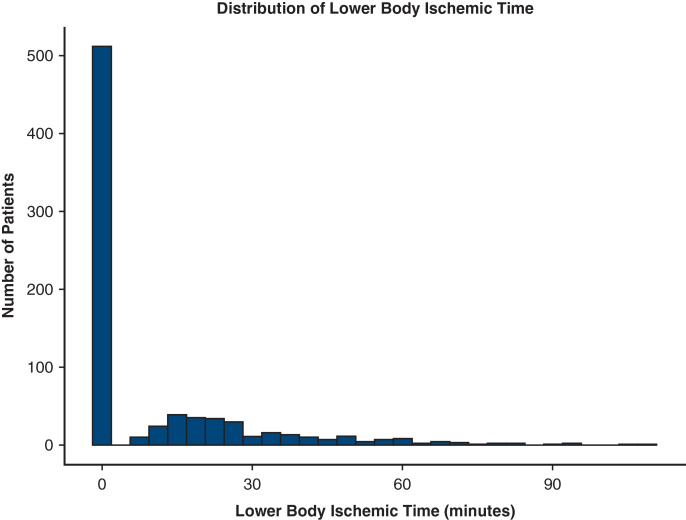
Histogram of LBI duration. Includes patients who did not undergo circulatory arrest (n = 357).

Operative characteristics are described in Table 3. The aorta was the most common site of arterial cannulation in both patients with POH and patients without (89.9% vs 91.9%, *P* = .213). The median nadir temperature for both groups was 31.9 °C (*P* = .705) with similar rates of CA (38.8% vs 35.9%, *P* = .437) including 128 patients without hypoxemia and 168 with hypoxemia. Median LBI duration was comparable in both cohorts (24.5 vs 21.5 minutes, *P* = .063). In the zero-inflated sample of all patients (Figure E3), 272 (62.8%) versus 234 (65.5%) patients had LBI of <1 minute, 118 (27.3%) versus 100 (28.0%) patients had LBI of 1-40 minutes, and 43 (9.9%) patients versus 23 (6.4%) patients had LBI greater than 40 minutes. Patients with and without POH had a similar incidence of intraoperative transfusion (69.5% vs 65.5%, *P* = .267).

**Table 3:** Operative characteristics

Variable	Overall, n = 790	No hypoxemia, n = 357	Hypoxemia, n = 433	*P* value
Intraoperative details				
Arterial cannulation site				.213
Aortic, n (%)	717 (90.8)	328 (91.9)	389 (89.9)	
Axillary, n (%)	38 (4.8)	12 (3.4)	26 (6.0)	
Other, n (%)	35 (4.4)	17 (4.8)	18 (4.2)	
Nadir temperature, °C, median [IQR]	31.90 [27.90, 32.20]	31.90 [27.20, 32.20]	31.90 [28.00, 32.20]	.705
Operative duration, min, median [IQR]	457.00 [391.00, 546.00]	442.00 [381.00, 538.00]	465.00 [398.00, 553.00]	.091
Circulatory arrest, n (%)	296 (37.5)	128 (35.9)	168 (38.8)	.437
Cerebral perfusion				.070
Antegrade, n (%)	171 (21.6)	66 (18.5)	105 (24.2)	
Retrograde, n (%)	111 (14.1)	58 (16.2)	53 (12.2)	
Cerebral perfusion time, min, median [IQR]	17.00 [10.12, 47.00]	16.00 [9.00, 40.25]	18.00 [12.00, 50.75]	.158
LBI				
LBI duration, min, n = 296, median [IQR]	23.00 [15.00, 37.00]	21.50 [14.00, 31.25]	24.50 [16.00, 40.00]	.063
<1 min, n (%)	506 (64.1)	234 (65.5)	272 (62.8)	.210
1-40 min, n (%)	218 (27.6)	100 (28.0)	118 (27.3)	
>40 min, n (%)	66 (8.4)	23 (6.4)	43 (9.9)	
Crossclamp time, min, median [IQR]	118.00 [89.25, 147.00]	116.00 [89.00, 145.00]	123.00 [90.00, 148.00]	.417
Cardiopulmonary bypass time, min, median [IQR]	154.50 [123.00, 193.00]	150.00 [120.00, 189.00]	159.00 [124.00, 198.00]	.117
Intraoperative transfusion, n (%)	535 (67.7)	234 (65.5)	301 (69.5)	.267

The overall cohort is presented and then divided into no hypoxemia vs hypoxemia (PF <300) groups. *IQR*, Interquartile range; *LBI*, lower-body ischemia; *PF*, arterial oxygen tension/inspired oxygen fraction ratio.

### Comparing No Hypoxemia Versus Hypoxemia Groups: Unweighted

There were 433 (54.8%) patients who developed POH, including 266 (61.4%) mild, 147 (33.9%) moderate, and 20 (4.6%) severe cases of POH respectively, whereas 357 (45.2%) patients did not develop POH. In an unweighted comparison of outcomes (Table E1) across none, mild, and moderate/severe hypoxemia groups, there was a stepwise increase in total ventilation hours (median 8.00 vs 9.18 vs 11.8 hours, *P* = .002); duration of supplemental oxygen use (104.75 vs 123.42 vs 132.58 hours, *P* < .001); ICU hours (49.85 vs 69.50 vs 85.75 hours, *P* < .001); and incidence of pneumonia (2.8% vs 4.5% vs 10.2%, *P* = .001). In total, 396 (50.1%) patients met criteria for the composite respiratory morbidity end point, including 154 (43.1%) patients without POH, 143 (53.8%) patients with mild POH, and 99 (59.3%) patients with moderate/severe POH. There were relatively few incidences of operative mortality overall (n = 18, 2.3%).

### Comparing No Hypoxemia Versus Hypoxemia Groups: IPTW

IPTW created well-balanced cohorts of 146.72 (effectively 147) patients with no, 149.31 (149) mild, and 145.90 (146) moderate/severe hypoxemia, respectively (Table E2). Weighted in-hospital outcomes are documented in Table 4. In this balanced cohort, there was no difference in operative mortality (2.5% vs 1.5% vs 2.9%, *P* = .637). There was a significant stepwise increase in the end point of composite respiratory morbidity (43.8% vs 50.6% vs 57.0%, *P* = .030). In addition, there remained a stepwise increase in the duration of supplemental oxygen use (105.00 vs 118.23 vs 123.83 hours, *P* = .010) and ICU hours (49.85 vs 65.70 vs 82.00 hours, *P* < .001), alongside a stepwise increase in the rate of ICU readmission (2.5% vs 3.6% vs 11.4%, *P* = .019). Although there was a similar stepwise increase in rates of reintubation (4.4% vs 4.7% vs 6.9%), this difference did not meet significance (*P* = .421). There remained an association between total ventilation hours (median 8.15 vs 8.05 vs 11.00 hours, *P* = .046) and postoperative pneumonia and severity of POH (4.2% vs 3.6% vs 9.6%, *P* = .019).

**Table 4:** Weighted in-hospital outcomes

Variable	None, PF >300 (n = 146.72)	Any hypoxemia (n ≈ 295)		*P* value (*P* < .05)
		Mild, PF 300-200 (n = 149.31)	Moderate/severe, PF ≤200 (n = 145.90)	Significant groups
Operative mortality, n (%)	3.6 (2.5)	2.3 (1.5)	4.3 (2.9)	.637
Composite respiratory morbidity, n (%)∗	64.2 (43.8)	75.6 (50.6)	83.1 (57.0)	**.030** (none vs moderate/severe)
Postoperative transfusion, n (%)	44.1 (30.0)	38.3 (25.7)	36.6 (25.1)	.461
Total ventilation hours, median [IQR]	8.15 [5.17, 14.45]	8.05 [5.00, 16.00]	11.00 [5.05, 25.00]	**.046** (none vs moderate/severe)
Reintubation, n (%)	6.4 (4.4)	7.0 (4.7)	10.0 (6.9)	.421
Supplemental oxygen duration, h, median [IQR]	105.00 [75.02, 170.33]	118.23 [83.68, 190.27]	123.83 [87.10, 214.08]	**.010** (none vs moderate/severe)
Return to the operating room				
Rebleeding, n (%)	3.8 (2.6)	3.7 (2.5)	9.0 (6.2)	.058
Other, n (%)	4.9 (3.3)	4.1 (2.8)	9.7 (6.7)	.104
ICU hours (total), median [IQR]	49.85 [27.83, 104.27]	65.70 [25.62, 118.00]	82.00 [42.00, 152.00]	**<.001** (none vs moderate/severe, mild vs moderate/severe)
ICU readmission, n (%)	3.6 (2.5)	5.4 (3.6)	11.4 (7.8)	**.019** (none vs moderate/severe)
Sternal wound infection, n (%)	2.6 (1.7)	1.7 (1.1)	0.0 (0.0)	.234
Pneumonia, n (%)	6.2 (4.2)	5.4 (3.6)	14.0 (9.6)	**.019**
Effusion, n (%)	5.1 (3.5)	6.1 (4.1)	6.0 (4.1)	.929
Stroke, n (%)	4.6 (3.1)	5.0 (3.3)	5.9 (4.0)	.865
Length of stay, d, median [IQR]	7.00 [6.00, 10.00]	7.00 [5.00, 10.00]	8.00 [5.00, 12.00]	.388

After the application of inverse probability of treatment weighting, the overall cohort was divided into no hypoxemia (“none”) versus mild hypoxemia versus moderate/severe hypoxemia. For all *P* < .05, Bonferroni correction was performed, and significant subgroups were noted. Values in bold indicate significant *P* values. *PF*, Arterial oxygen tension/inspired oxygen fraction ratio; *IQR*, interquartile range; *ICU*, intensive care unit.

∗ Composite respiratory morbidity defined as at least 1 of the following: prolonged invasive ventilation (Society of Thoracic Surgeons definition ≥24 hours), supplemental oxygen use ≥120 hours, reintubation, or postoperative pneumonia.

### Influence of POH on Survival

**Figure 1:**
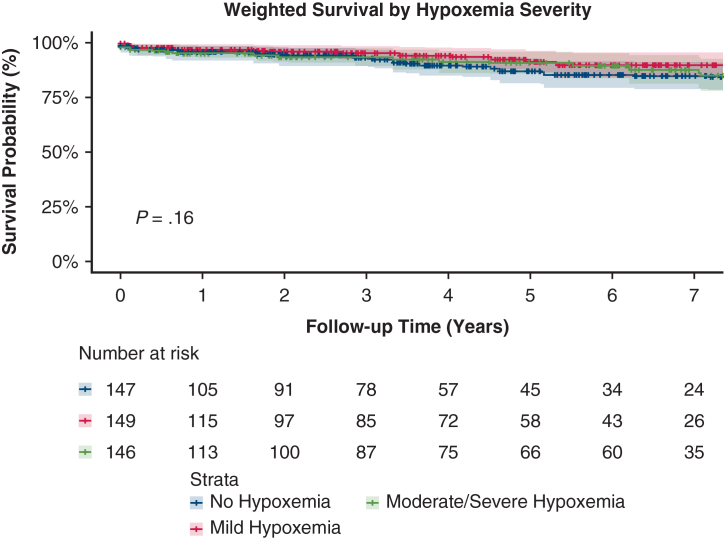
Balanced (inverse probability of treatment weighting) Kaplan–Meier curve for the association between severity of postoperative hypoxemia and all-cause mortality. 95% CI was used.

There were 63 (8.0%) deaths within 7 years. IPTW-weighted Kaplan-Meier (Figure 1) demonstrated no significant difference in survival by POH severity (84.8% vs 89.8% vs 87.6%, *P* = .160) within 7 years. In Cox proportional hazards models restricted to preoperative and intraoperative covariates, POH was not associated with 7-year all-cause mortality (hazards ratio [HR], 0.76; 95% CI, 0.43-1.33; *P* = .328). Results were materially unchanged when additionally adjusting for postoperative complications (HR, 0.72; 95% CI, 0.38-1.37, *P* = .321; Table 5). In the complication-adjusted model, postoperative transfusion (HR, 2.72; 95% CI, 1.41-5.24; *P* = .003) was the only variable associated with 7-year mortality, whereas nadir temperature (HR, 1.00; 95% CI, 0.94-1.06; *P* = .920); sternal wound infection (HR, 0.23; 95% CI, 0.01-3.68; *P* = .297); pneumonia (HR, 2.11; 95% CI, 0.87-5.12; *P* = .097), stroke (HR, 3.16; 95% CI, 0.97-10.27; *P* = .056); and length of stay (HR, 1.01; 95% CI, 0.99-1.04; *P* = .287) were not.

**Table 5:** Weighted multivariable Cox proportional hazard ratio for all-cause mortality within 7 years

Variables	Hazard ratio (95% CI)	*P* value (*P* < .05)
Hypoxemia	0.72 (0.38-1.37)	.321
Nadir temperature, °C	1.00 (0.94-1.06)	.920
Postoperative transfusion	2.72 (1.41-5.24)	**.003**
Sternal wound infection	0.23 (0.01-3.68)	.297
Pneumonia	2.11 (0.87-5.12)	.097
Stroke	3.16 (0.97-10.27)	.056
Length of stay, d	1.01 (0.99-1.04)	.287

Baseline characteristics and postoperative complications were included in Cox hazard analysis with end point for all-cause mortality within 7 years. Variables were selected on the basis of clinical grounds and from univariate screens (*P* < .10). Values in bold indicate significant *P* values.

### Relationship Between CA and POH

In a weighted multivariable logistic regression (Table 6), the use of CA was not associated with the development of moderate/severe hypoxemia (odds ratio [OR], 0.96; 95% CI, 0.60-1.53; *P* = .864) and neither was nadir temperature (OR, 0.98; 95% CI, 0.93-1.04], *P* = .492) or increasing intraoperative pRBC units (OR, 1.01; 95% CI, 0.91-1.13; *P* = .814).

**Table 6:** Weighted multivariable logistic regression for development of moderate/severe hypoxemia

Variables	Odds ratio (95% CI)	*P* value (*P* < .05)
Circulatory arrest	0.96 (0.60-1.53)	.864
Nadir temperature, °C	0.98 (0.93-1.04)	.492
Intraoperative pRBC units	1.01 (0.91-1.13)	.814

Baseline and perioperative characteristics were included in logistic regression for the development of moderate/severe hypoxemia (PF <200) after inverse probability of treatment weighting. Variables were selected on the basis of clinical grounds and from univariate screens (*P* < .10). *pRBC*, Packed red blood cells; *PF*, arterial oxygen tension/inspired oxygen fraction ratio.

**Figure E4:**
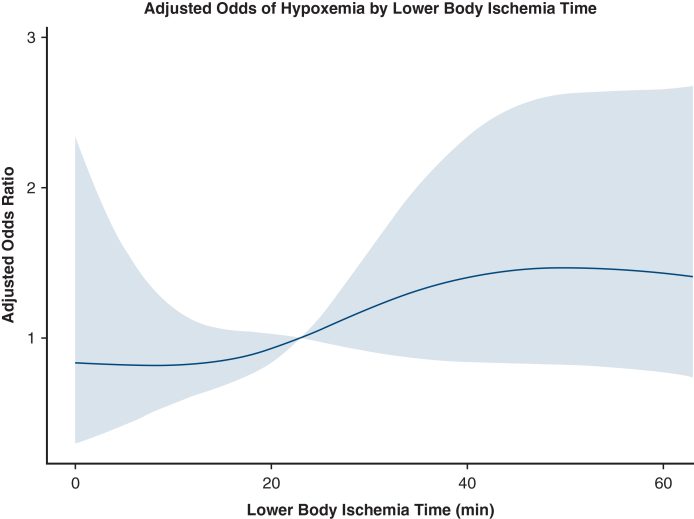
Adjusted regression cubic spline of only circulatory arrest patients. A cubic spline with knots of 4 was used to assess the potentially nonlinear relationship between circulatory arrest time and development any hypoxemia (arterial oxygen tension/inspired oxygen fraction ratio <300) at 6 hours postoperatively, while adjusting for covariates including age, sex at birth, lifetime smoking, chronic obstructive pulmonary disease, and cardiopulmonary bypass time.

The granular relationship between LBI duration and development of any hypoxemia was also investigated with a cubic spline (Figure E4). LBI duration demonstrated a trend toward positive association with the odds of developing any hypoxemia in the adjusted spline, although there was a wide CI throughout the curve.

### Subgroup Analysis: CA-Only Cohort

In an IPTW-weighted (Table E3) multivariable logistic regression of only patients who underwent CA (Table E4), none of the investigated predictors, including LBI time (OR, 1.00; CI, 0.98-1.02; *P* = .723), was associated with the development of moderate/severe hypoxemia. Similarly, a weighted multivariable linear regression did not identify LBI time as associated with postoperative PF ratio at 6 hours (Table E5).

### Sensitivity Analyses

Covariates with residual imbalance in the 0.10 to 0.20 range were evaluated in sensitivity analyses using a doubly robust approach in which these variables were additionally included in IPTW-weighted regression models. The inclusion of these covariates did not materially alter effect estimates or statistical inferences.

## Discussion

**Figure 2:**
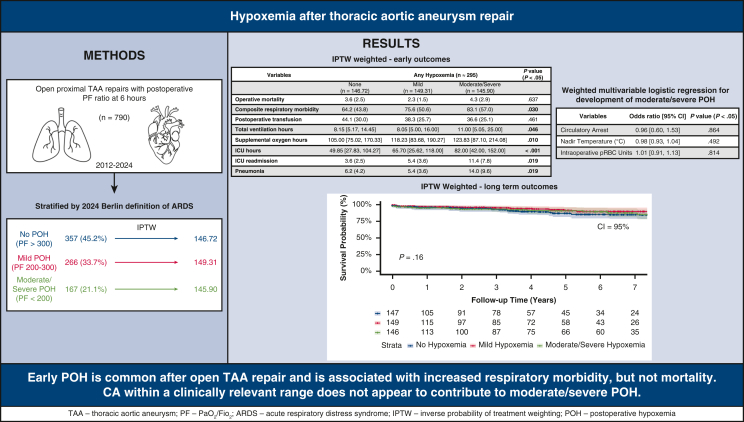
Early POH (PF ratio at 6 hours) is common after open thoracic aortic aneurysm repair. After IPTW, increasing hypoxemia severity is associated with greater morbidity but not early mortality or long-term survival. Circulatory arrest and clinically typical arrest durations do not appear to meaningfully contribute to moderate or severe hypoxemia (PF < 200).

To our knowledge, contemporary data examining graded POH using standardized 6-hour PF ratio criteria in patients undergoing thoracic aortic aneurysm repair remain limited. In this cohort, we demonstrate that POH at 6 hours is associated with increased respiratory morbidity. We also found no obvious association between 6-hour POH and need of CA (Figure 2).

In this contemporary cohort, POH was common, affecting more than one half of our patients, although the majority of cases were mild in severity. This aligns with previous reports following complex cardiac and aortic surgery; POH is a frequent—but often limited—early postoperative phenomenon.^1^, ^2^, ^3^ Across both unadjusted and weighted analyses, POH was consistently associated with markers of increased respiratory morbidity, including prolonged mechanical ventilation and ICU stays. These relationships are well documented in the current literature.^2^^,^^4^^,^^5^ Importantly, moderate-to-severe POH was also associated with postoperative pneumonia, a complication that independently predicted 7-year all-cause mortality in a CA only cohort when hypoxemia did not. Taken together, these findings suggest that hypoxemia may function both as a marker of pulmonary vulnerability and an indirect driver of mortality, with downstream complications such as pneumonia mediating adverse long-term outcomes. Already widely solidified in the literature across uni- and multicenter studies, pneumonia confers a significant risk of mortality in the cardiac surgery population.^31^, ^32^, ^33^ We did not observe a survival difference by hypoxemia severity in the overall cohort. Rather, other postoperative pulmonary complications, particularly pneumonia, may better explain long-term differences.^34^ In addition, although CA did not appear to drive early hypoxemia, the long-term systemic consequences of the physiologic stress it imposes—particularly in vulnerable subgroups—remain incompletely understood and warrant further study.^8^^,^^35^ More broadly, whether POH represents a modifiable therapeutic target or primarily reflects the magnitude of underlying surgical and inflammatory stress remains unclear. Prospective investigations evaluating targeted strategies to maintain normoxemia after thoracic aortic surgery may help clarify whether intervention can meaningfully reduce postoperative morbidity or whether hypoxemia serves principally as a marker of overall physiologic insult.

In the present study, the use of CA was not associated with the development of moderate-to-severe hypoxemia. Consistent with this, in a subanalysis limited to patients undergoing CA, LBI duration was not associated with the development of either moderate-to-severe hypoxemia or PF ratio at 6 hours, reinforcing the limited impact of arrest on early postoperative oxygenation. Taken together, LBI duration within a clinically relevant range did not provide a meaningful contribution to POH in our cohort. Instead, any intraoperative contribution to POH may relate primarily to the systemic inflammatory and pulmonary effects of CPB, as suggested in prior literature.^8^^,^^9^ This stands out from the ATAAD hypoxemia literature, where although conclusions on the use of CA is varied,^15^^,^^36^ CA duration is consistently identified as an independent risk factor for POH.^1^^,^^13^^,^^20^^,^^37^ Specifically, Shen and colleagues^37^ found that duration of CA had an OR of 1.123 per minute increment of resulting in postoperative oxygenation impairment, whereas Liu and colleagues^1^ identified deep hypothermic CA time >25 minutes as an independent predictor of POH at 72 hours. Although prior reports have suggested that hypothermia strategy may influence pulmonary outcomes, nadir temperature was not independently associated with POH or long-term mortality in our weighted analyses. A plausible explanation for these discrepant findings may lie in fundamental differences in disease state instead of the practice of CA per se. Patients with ATAAD frequently present with impaired baseline oxygenation, greater physiological stress, and increased surgical complexity, factors that may amplify the pulmonary impact of prolonged CA as compared with aneurysm and chronic dissection repair.^10^^,^^38^^,^^39^

### Limitations

There are several limitations in our study. First, this is a single-center retrospective study, limiting the reproducibility and generalizability due to unique institutional perfusion and ICU practices. Although our main analysis may be considered robust, our subgroup analysis, notably after weighting, was limited by a smaller number of patients who underwent CA. Second, our study uses a PF ratio of <200 at 6 ± 3 hours, which accounts for a specific window of hypoxemia, although transient hypoxemia may have occurred before or set in after this period. Third, PF ratio is influenced by ventilator settings and documentation of Fio_2_/positive end-expiratory pressure, which may vary across clinicians and could lead to misclassification of hypoxemia severity. Fourth, although IPTW improved balance in measured covariates, residual confounding is possible, given the nonrandom selection of CA and unmeasured intraoperative and ICU factors (eg, ventilation strategy, fluid/transfusion practices). Finally, management strategies evolved over the 2012-2024 study period, and secular changes in perfusion and ICU protocols may have influenced both exposure selection and postoperative oxygenation.

## Conclusions

In our study, we found a significant association between severity of POH and respiratory morbidity. Additionally, our results support the pulmonary safety of CA in thoracic aortic aneurysm surgery.

## Conflict of Interest Statement

H.T. reported consultant for Artivion and Edwards Lifesciences. All other authors reported no conflicts of interest.

The *Journal* policy requires editors and reviewers to disclose conflicts of interest and to decline handling or reviewing manuscripts for which they may have a conflict of interest. The editors and reviewers of this article have no conflicts of interest.

## Figures and Tables

**Table E1:** Unadjusted in-hospital outcomes

Variable	Overall (n = 790)	None, PF >300 (n = 357)	Any hypoxemia (n = 433)		*P* value (*P* < .05)
			Mild, PF 300-200 (n = 266)	Moderate/severe, PF ≤ 200 (n = 167)	Significant groups
Operative mortality, n (%)	18 (2.3)	7 (2.0)	6 (2.3)	5 (3.0)	.761
Composite respiratory morbidity, n (%)∗	396 (50.1)	154 (43.1)	143 (53.8)	99 (59.3)	**.001** (none vs mild, none vs moderate/severe)
Postoperative transfusion, n (%)	222 (28.1)	105 (29.4)	75 (28.2)	42 (25.1)	.599
Total ventilation hours, median [IQR]	9.00 [5.18, 17.38]	8.00 [5.17, 14.00]	9.18 [5.07, 17.97]	11.80 [5.48, 36.79]	**.002** (none vs moderate/severe)
Reintubation, n (%)	50 (6.3)	19 (5.3)	18 (6.8)	13 (7.8)	.524
Supplemental oxygen duration, h, median [IQR]	114.32 [81.54, 192.57]	104.75 [75.02, 155.95]	123.42 [86.58, 196.62]	132.58 [88.15, 223.42]	**<.001** (none vs mild, none vs moderate/severe)
Return to the operating room					
Rebleeding, n (%)	32 (4.1)	10 (2.8)	11 (4.1)	11 (6.6)	.122
Other, n (%)	30 (3.8)	11 (3.1)	8 (3.0)	11 (6.6)	.105
ICU hours, total, median [IQR]	65.31 [32.00, 119.24]	49.85 [30.30, 97.00]	69.50 [28.18, 125.00]	85.75 [43.69, 156.09]	**<.001** (none vs moderate/severe)
ICU readmission, n (%)	34 (4.3)	10 (2.8)	12 (4.5)	12 (7.2)	.069
Sternal wound infection, n (%)	6 (0.8)	3 (0.8)	3 (1.1)	0 (0.0)	.430
Pneumonia, n (%)	39 (4.9)	10 (2.8)	12 (4.5)	17 (10.2)	**.001** (none vs moderate/severe)
Effusion, n (%)	34 (4.3)	18 (5.0)	10 (3.8)	6 (3.6)	.648
Stroke, n (%)	28 (3.5)	9 (2.5)	11 (4.1)	8 (4.8)	.346
Length of stay, d, median [IQR]	7 [6, 10]	7 [5, 9]	7 [6, 11]	8 [6, 12]	.055

The overall cohort was divided into no hypoxemia (“none”) versus mild hypoxemia versus moderate/severe hypoxemia. For all *P* < .05, Bonferroni correction was performed, and significant subgroups were noted. Values in bold indicate significant *P* values. *PF*, Arterial oxygen tension/inspired oxygen fraction ratio; *IQR*, interquartile range; *ICU*, intensive care unit.

∗ Composite respiratory morbidity defined as at least 1 of the following: prolonged invasive ventilation (Society of Thoracic Surgeons definition ≥24 hours), supplemental oxygen use ≥120 hours, reintubation, or postoperative pneumonia.

**Table E2:** Three-way inverse probability of treatment weighting

Variable	None, n = 146.72	Mild hypoxemia, n = 149.31	Moderate/severe hypoxemia, n = 145.90	*P* value	SMD
Demographics					
Mean age (y), median [IQR]	63.00 [54.00, 70.00]	63.21 [54.00, 71.00]	61.00 [53.00, 71.00]	.955	0.015
Female, n (%)	21.3 (14.5)	25.0 (16.8)	25.1 (17.2)	.711	0.048
BMI, kg/m^2^, median [IQR]	28.39 [25.83, 32.40]	29.07 [25.88, 32.37]	28.11 [26.01, 32.64]	.894	0.010
Non-White, n (%)	27.3 (18.6)	28.6 (19.2)	21.9 (15.0)	.481	0.074
Hispanic/Latino ethnicity, n (%)	24.7 (16.8)	24.3 (16.2)	23.6 (16.2)	.979	0.012
Smoker, n (%)	64.5 (44.0)	62.9 (42.1)	62.0 (42.5)	.922	0.024
Emergent status, n (%)	1.8 (1.2)	0.7 (0.5)	1.0 (0.7)	.696	0.055
Risk factors					
Hypertension, n (%)	115.5 (78.7)	117.3 (78.6)	114.5 (78.5)	.998	0.004
Diabetes, n (%)	23.1 (15.8)	22.8 (15.3)	20.4 (14.0)	.875	0.033
Atrial fibrillation, n (%)	29.6 (20.2)	31.4 (21.1)	31.7 (21.7)	.933	0.025
Connective tissue disease, n (%)	1.6 (1.1)	1.0 (0.6)	0.0 (0.0)	.257	0.102
Coronary artery disease, n (%)	124.2 (84.6)	126.7 (84.9)	121.1 (83.0)	.849	0.034
Peripheral artery disease, n (%)	25.9 (17.7)	23.8 (15.9)	27.5 (18.9)	.732	0.052
CKD, n (%)	30.7 (20.9)	25.6 (17.2)	25.0 (17.1)	.537	0.065
eGFR, mL/min/1.73 m^2^, median [IQR]	79.93 [65.03, 92.00]	82.33 [65.53, 93.85]	79.22 [67.34, 90.04]	.611	0.031
COPD, n (%)	17.1 (11.7)	16.6 (11.1)	16.6 (11.4)	.984	0.012
CVD, n (%)	13.5 (9.2)	14.5 (9.7)	18.1 (12.4)	.505	0.069
MI, n (%)	8.6 (5.9)	10.3 (6.9)	9.0 (6.2)	.907	0.028
CVA, n (%)	9.5 (6.5)	7.7 (5.2)	7.6 (5.2)	.798	0.038
CHF, n (%)	59.9 (40.8)	59.0 (39.5)	63.2 (43.3)	.728	0.051
Ejection fraction, median [IQR]	55.00 [55.00, 60.00]	55.00 [50.00, 60.05]	55.00 [53.00, 60.00]	.481	0.092
Reintervention, n (%)	44.2 (30.2)	45.5 (30.5)	43.4 (29.7)	.986	0.011
Aortic aneurysm, n (%)	143.1 (97.5)	147.3 (98.6)	143.7 (98.5)	.530	0.055
Laboratory test results					
Hemoglobin, median [IQR]	14.00 [13.00, 14.90]	14.00 [12.90, 15.00]	14.20 [12.81, 15.00]	.858	0.047
WBC count, median [IQR]	6.82 [5.38, 8.14]	6.95 [5.42, 8.40]	6.70 [5.75, 7.96]	.567	0.074
Platelet count, median [IQR]	205.00 [172.00, 243.00]	200.70 [167.00, 246.58]	199.00 [172.00, 248.00]	.874	0.049
Albumin, median [IQR]	4.40 [4.10, 4.70]	4.50 [4.20, 4.70]	4.50 [4.20, 4.70]	.263	0.083
Hematocrit, median [IQR]	41.80 [38.87, 44.10]	42.00 [39.20, 44.30]	42.10 [38.30, 44.62]	.628	0.048
Creatinine, median [IQR]	1.00 [0.88, 1.20]	1.00 [0.86, 1.16]	1.01 [0.88, 1.15]	.759	0.026
Procedures					
Isolated ascending aorta replacement, n (%)	18.6 (12.7)	18.4 (12.4)	18.8 (12.9)	.987	0.011
Concomitant AVR/r w/o ARR/r, n (%)	20.0 (13.6)	19.8 (13.2)	20.7 (14.2)	.959	0.018
Aortic root replacement/repair, n (%)	66.0 (45.0)	65.8 (44.1)	64.0 (43.9)	.972	0.014
Aortic arch replacement, n (%)	52.5 (35.8)	48.3 (32.4)	47.7 (32.7)	.713	0.048
Concomitant procedure, n (%)	27.2 (18.5)	29.1 (19.5)	28.2 (19.4)	.966	0.016
Intraoperative details					
Nonaortic arterial cannulation site, n (%)	16.4 (11.2)	14.4 (9.7)	14.1 (9.6)	.843	0.034
Operative duration, min, median [IQR]	440.00 [380.72, 529.00]	455.89 [392.27, 542.45]	445.00 [381.00, 512.00]	.482	0.053
Crossclamp time, min, median [IQR]	116.00 [89.00, 143.00]	117.00 [83.20, 145.00]	113.34 [88.33, 144.00]	.885	0.063
Cardiopulmonary bypass time, min, median [IQR]	150.00 [120.00, 186.00]	151.76 [120.24, 188.00]	151.00 [121.27, 175.45]	.933	0.018
Intraoperative pRBC transfusion, n (%)	33.4 (22.8)	31.7 (21.2)	31.5 (21.6)	.918	0.024

The overall cohort was divided into no hypoxemia (“none”) vs mild hypoxemia vs moderate/severe hypoxemia before inverse probability of treatment weighting. SMD < 0.1 was considered well balanced. *SMD*, Standardized mean difference; *IQR*, interquartile range; *BMI*, body mass index; *CKD*, chronic kidney disease; *eGFR*, estimated glomerular filtration rate; *COPD*, chronic obstructive pulmonary disease; *CVD*, cardiovascular disease; *MI*, myocardial infarction; *CVA*, cerebrovascular accident; *CHF*, congestive heart failure; *WBC*, white blood cell; *AVR/r*, aortic valve replacement/repair; *ARR/r*, aortic root replacement/repair; *pRBC*, packed red blood cell.

**Table E3:** Subgroup analysis—3-way inverse probability of treatment weighting

Variable	None, n = 42.74	Mild hypoxemia, n = 42.28	Moderate/severe hypoxemia, n = 40.41	*P* value	SMD
Demographics					
Mean age, y, median [IQR]	63.00 [53.22, 72.00]	61.26 [54.00, 71.00]	62.62 [53.50, 70.57]	.893	0.045
Female, n (%)	8.2 (19.2)	8.7 (20.6)	7.9 (19.5)	.977	0.024
BMI, kg/m^2^, median [IQR]	29.41 [25.61, 32.42]	29.03 [24.76, 32.09]	27.03 [25.67, 33.39]	.854	0.043
Non-White, n (%)	4.9 (11.4)	5.8 (13.8)	6.0 (14.8)	.808	0.067
Hispanic/Latino ethnicity, n (%)	7.2 (16.9)	7.0 (16.7)	8.2 (20.2)	.853	0.061
Smoker, n (%)	22.4 (52.4)	19.3 (45.7)	19.3 (47.8)	.746	0.089
Emergent status, n (%)	0.0 (0.0)	0.0 (0.0)	0.0 (0.0)	.362	<0.001
Risk factors					
Hypertension, n (%)	33.2 (77.7)	34.1 (80.7)	31.5 (77.9)	.898	0.049
Diabetes, n (%)	7.1 (16.6)	6.8 (16.2)	5.7 (14.1)	.921	0.047
Atrial fibrillation, n (%)	8.9 (20.9)	8.4 (19.9)	8.1 (20.0)	.988	0.017
Connective tissue disease, n (%)	0.9 (2.2)	0.3 (0.7)	0.0 (0.0)	.393	0.151
Coronary artery disease, n (%)	35.1 (82.1)	33.6 (79.5)	33.5 (82.9)	.885	0.058
Peripheral artery disease, n (%)	7.7 (18.0)	8.6 (20.3)	9.5 (23.5)	.739	0.091
CKD, n (%)	6.6 (15.5)	5.2 (12.3)	5.5 (13.7)	.873	0.062
eGFR, mL/min/1.73 m^2^, median [IQR]	79.82 [67.99, 89.46]	83.32 [72.87, 95.14]	82.76 [70.42, 90.54]	.422	0.064
COPD, n (%)	6.8 (15.9)	5.8 (13.6)	5.7 (14.1)	.929	0.043
CVD, n (%)	5.5 (12.8)	5.8 (13.7)	7.1 (17.6)	.713	0.091
MI, n (%)	1.0 (2.3)	2.2 (5.2)	1.7 (4.3)	.643	0.101
CVA, n (%)	3.4 (8.0)	2.8 (6.7)	3.3 (8.1)	.944	0.036
CHF, n (%)	16.0 (37.3)	15.5 (36.6)	11.5 (28.4)	.527	0.127
Ejection fraction, median [IQR]	55.00 [54.72, 60.00]	58.00 [53.00, 63.00]	55.37 [53.00, 59.25]	.687	0.092
Reintervention, n (%)	14.8 (34.6)	16.0 (37.9)	15.0 (37.0)	.923	0.046
Aortic aneurysm, n (%)	41.6 (97.3)	40.7 (96.3)	38.9 (96.3)	.894	0.040
Laboratory test results					
Hemoglobin, median [IQR]	13.66 [12.70, 14.60]	13.80 [12.80, 15.16]	13.50 [12.30, 14.90]	.716	0.079
WBC Count, median [IQR]	6.70 [5.24, 8.12]	7.00 [5.88, 8.64]	7.08 [5.58, 8.28]	.442	0.148
Platelet Count, median [IQR]	203.37 [169.47, 235.41]	200.76 [170.02, 239.88]	209.00 [168.52, 257.06]	.583	0.115
Albumin, median [IQR]	4.40 [4.00, 4.68]	4.40 [4.10, 4.60]	4.40 [4.13, 4.70]	.891	0.031
Hematocrit, median [IQR]	40.29 [38.84, 43.60]	41.74 [38.70, 45.00]	41.63 [37.11, 44.30]	.564	0.138
Creatinine, median [IQR]	1.00 [0.89, 1.09]	0.99 [0.86, 1.10]	0.99 [0.83, 1.15]	.677	0.089
Procedures					
Isolated ascending aorta replacement, n (%)	0.2 (0.5)	0.5 (1.2)	1.0 (2.5)	.481	0.109
Concomitant AVR/r w/o ARR/r, n (%)	5.4 (12.6)	5.0 (11.8)	5.6 (13.7)	.953	0.038
Aortic root replacement/repair, n (%)	25.5 (59.7)	23.5 (55.5)	24.2 (59.8)	.857	0.058
Aortic arch replacement, n (%)	38.5 (90.1)	37.9 (89.7)	36.8 (91.1)	.963	0.032
Concomitant procedure, n (%)	4.8 (11.2)	5.4 (12.9)	6.2 (15.5)	.755	0.084
Intraoperative details					
Nonaortic arterial cannulation site, n (%)	9.2 (21.5)	9.9 (23.5)	9.7 (23.9)	.944	0.038
Operative duration, min, median [IQR]	476.48 [405.67, 608.52]	501.89 [433.00, 588.18]	468.46 [417.57, 557.88]	.596	0.056
Crossclamp time, min, median [IQR]	118.00 [79.45, 147.00]	131.00 [80.00, 158.00]	122.56 [76.00, 150.00]	.767	0.057
Cardiopulmonary bypass time, min, median [IQR]	177.00 [138.00, 211.00]	178.27 [149.07, 211.80]	171.77 [140.00, 203.63]	.893	0.062
Intraoperative pRBC transfusion, n (%)	17.2 (40.3)	16.0 (37.9)	15.8 (39.2)	.963	0.032

The subgroup was divided into no hypoxemia (“none”) vs mild hypoxemia vs moderate/severe hypoxemia before inverse probability of treatment weighting. SMD < 0.1 was considered well balanced; however, given limited power, residual imbalance (SMD 0.10-0.20) was tolerated for covariates judged unlikely to materially influence outcomes. *SMD*, Standardized mean difference; *IQR*, interquartile range; *BMI*, body mass index; *CKD*, chronic kidney disease; *eGFR*, estimated glomerular filtration rate; *COPD*, chronic obstructive pulmonary disease; *CVD*, cardiovascular disease; *MI*, myocardial infarction; *CVA*, cerebrovascular accident; *CHF*, congestive heart failure; *WBC*, white blood cell; *AVR/r*, aortic valve replacement/repair; *ARR/r*, aortic root replacement/repair; *pRBC*, packed red blood cell.

**Table E4:** Subgroup analysis—weighted multivariable logistic regression for development of moderate/severe hypoxemia

Variables	Odds ratio (95% CI)	*P* value (*P* < .05)
Lower body ischemia time, min	1.00 (0.98, 1.02)	.723
Nadir temperature, °C	1.00 (0.91, 1.09)	.952
Intraoperative pRBC units	1.04 (0.89, 1.20)	.636

Baseline and perioperative characteristics for the subgroup were included in logistic regression for the development of moderate/severe hypoxemia (arterial oxygen tension/inspired oxygen fraction ratio <200) after inverse probability of treatment weighting. Variables were selected on the basis of clinical grounds and from univariate screens (*P* < .10). *pRBC*, Packed red blood cell.

**Table E5:** Subgroup analysis—weighted multivariable linear regression for PF ratio at 6 hours

Variables	*β* coefficient (95% CI)	*P* value (*P* < .05)
Lower body ischemia time, min	−0.33 (−1.11 to 0.46)	.410
Nadir temperature, °C	−0.40 (−4.12 to 3.33)	.833
Intraoperative pRBC units	−1.50 (−7.52 to 4.51)	.623

Baseline and perioperative characteristics for the subgroup were included in linear regression for arterial oxygen tension/inspired oxygen fraction ratio at 6 hours postoperatively after inverse probability of treatment weighting (IPTW). Variables were selected on the basis of clinical grounds and from univariate screens (*P* < .10). *PF*, Arterial oxygen tension/inspired oxygen fraction ratio; *pRBC*, packed red blood cell.
